# b-move: Faster Lossless Approximate Pattern Matching in a Run-Length Compressed Index

**DOI:** 10.21203/rs.3.rs-5367343/v1

**Published:** 2024-11-18

**Authors:** Lore Depuydt, Luca Renders, Simon Van de Vyver, Lennart Veys, Travis Gagie, Jan Fostier

**Affiliations:** Ghent University - imec, Technologiepark 126, 9052 Ghent, Belgium; Ghent University - imec, Technologiepark 126, 9052 Ghent, Belgium; Ghent University, Technologiepark 126, 9052 Ghent, Belgium; Ghent University, Technologiepark 126, 9052 Ghent, Belgium; Dalhousie University, 6050 University Avenue, PO BOX 15000, Halifax, NS B3H 4R2, Canada; Ghent University - imec, Technologiepark 126, 9052 Ghent, Belgium

**Keywords:** Pan-genomics, FM-index, r-index, Move Structure, Bidirectional Search, Approximate Pattern Matching, Lossless Alignment, Cache Efficiency

## Abstract

**Background:**

Due to the increasing availability of high-quality genome sequences, pan-genomes are gradually replacing single consensus reference genomes in many bioinformatics pipelines to better capture genetic diversity. Traditional bioinformatics tools using the FM-index face memory limitations with such large genome collections. Recent advancements in run-length compressed indices like Gagie et al.’s r-index and Nishimoto and Tabei’s move structure, alleviate memory constraints but focus primarily on backward search for MEM-finding. Arakawa et al.’s br-index initiates complete approximate pattern matching using bidirectional search in run-length compressed space, but with significant computational overhead due to complex memory access patterns.

**Results:**

We introduce b-move, a novel bidirectional extension of the move structure, enabling fast, cache-efficient, lossless approximate pattern matching in run-length compressed space. It achieves bidirectional character extensions up to 7 times faster than the br-index, closing the performance gap with FM-index-based alternatives. For locating occurrences, b-move performs ϕ and $\phi^{-1}$ operations up to 7 times faster than the br-index. At the same time, it maintains the favorable memory characteristics of the br-index, for example, all available complete *E. coli* genomes on NCBI’s RefSeq collection can be compiled into a b-move index that fits into the RAM of a typical laptop.

**Conclusions:**

b-move proves practical and scalable for pan-genome indexing and querying. We provide a C++ implementation of b-move, supporting efficient lossless approximate pattern matching including locate functionality, available at https://github.com/biointec/b-move under the AGPL-3.0 license.

## 1 Background

Since the advent of long-read sequencing platforms, the availability of high-quality genome sequences has increased dramatically. To exploit this data, it is becoming increasingly common to compile individuals from the same species or several related species into a single index, forming what is known as a pan-genome [1]. This approach aims to better capture genetic diversity and mitigate biased results stemming from the choice of reference.

Many widely used bioinformatics tools, such as BWA [2] and Bowtie 2 [3], rely on the FM-index [4]. The FM-index, based on the Burrows-Wheeler transform (BWT) [5] and suffix array (SA) [6], efficiently counts and locates exact occurrences of a search pattern in the reference. While it is compact and fast for relatively small references (e.g., a single human reference genome or a few dozen bacterial species), its memory use scales linearly with the total genome content. This limitation calls for new index types capable of storing and analyzing large genome collections within the memory constraints of modern workstations and laptops.

The BWT’s inherent compressibility [7] (see e.g., bzip2 [8]), particularly for highly repetitive input texts like pan-genomes, has led to a focus on run-length compression. The run-length FM-index (RLFM-index) [9] efficiently counts occurrences in O(r) space, r being the number of character runs in the BWT, but requires O(n) additional space for locating functionality. Recently, Gagie et al. [10, 11] introduced the r-index, which also supports locating functionality in O(r) space. Its reduced memory requirements have made the r-index the foundation for several tools, including MONI [12], PHONI [13], SPUMONI [14], and SPUMONI 2 [15]. Nishimoto and Tabei [16] more recently proposed the “move structure”, a run-length compressed index achieving, unlike the r-index, both O(r) space and O(1)-time LF operations. Built upon this, Movi [17] offers efficient pan-genome index building and querying, matching SPUMONI’s functionality but with significantly faster performance. Similarly, Move-r [18] provides a faster alternative to the r-index.

Despite these advancements, a notable limitation persists: aforementioned indexes and tools support only backward stepping through the LF operation, limiting the range of queries possible. Specifically, tools relying on the r-index or move structure focus almost exclusively on MEM-finding, using (pseudo-)matching lengths and statistics. In a pan-genome with thousands of genomes, this approach could yield an overwhelming number of MEMs, making downstream full read alignment based on seed-and-extend algorithms challenging and potentially infeasible [19].

Recognizing this limitation, Arakawa et al. [20] introduced the br-index. This extension of the traditional r-index enables bidirectional match extensions during the search process, i.e., both to the left and right, in arbitrary order. The br-index offers more operational flexibility, supporting functionalities like lossless approximate pattern matching (APM) based on the pigeonhole principle or more general search schemes [21]. In lossless approximate pattern matching, *all* occurrences in the search text within a certain maximum error distance of the query pattern, are guaranteed to be identified. On the downside, the br-index also inherits the high computational overhead of the r-index. This overhead stems from the intricate interplay of rank and select queries on compressed sparse bitvectors and wavelet trees, leading to multiple cache misses. Despite its favorable O(r) memory complexity, the br-index can be one order of magnitude slower than the bidirectional FM-index, hindering its adoption in practical bioinformatics tools.

### Contribution.

In this paper, we introduce b-move, a bidirectional extension of the move structure, as a faster alternative to the br-index. This paper is organized as follows. In Section 2, we recapitulate basic concepts and existing methods that form the foundation of this paper. In Section 3, we propose our bidirectional move structure, with a detailed description of its core bidirectional character extension, ϕ, and $\phi^{-1}$ functionalities. In Section 4, we demonstrate the efficiency of b-move in executing synchronized bidirectional character extensions, achieving speedups of 5 to 7 times compared to the br-index. For locating occurrences, we show that b-move performs ϕ and $\phi^{-1}$ operations up to 7 times faster than br-index. We observe that b-move performs comparably to FM-index-based tools while maintaining the br-index’s favorable O(r) memory complexity, which is superior to that of the bidirectional FM-index in both theory and practice. This paper extends our previous work presented at WABI 2024, where we introduced the initial version of b-move, which focused exclusively on bidirectional character extensions [22].

## 2 Preliminaries

In this paper, arrays and strings are indexed starting from zero. Consider a search text T with a length of n=|T| over an alphabet Σ. In the context of pan-genomes, T consists of multiple concatenated genome sequences. We assume that T ends with the sentinel character $, which is lexicographically smaller than any other character in Σ. A substring within string T is represented as an interval [i,j] over T, where 0≤i≤j≤n−1. The *i*th suffix of T, denoted as $T_{i}$, refers to the substring T[i,n−1].

In this paper, we primarily focus on accelerating two core elements of our approach to lossless approximate pattern matching: bidirectional character extensions, which are based on the LF operation, and locating occurrences using the ϕ and $\phi^{-1}$ operations. Consequently, our discussion will be limited to the data structures required for these two core operations and the computations needed to perform them.

### 2.1 Lossless approximate pattern matching

In lossless approximate pattern matching, all occurrences of a query pattern P within a text T are guaranteed to be identified, allowing for up to K errors according to Hamming (substitutions) or Levenshtein/edit (substitutions, insertions, and deletions) distance. This process, when based on an underlying unidirectional full-text index, is computationally demanding, especially for large error thresholds, due to the inefficiencies of naive backtracking [23].

To address this, Kucherov et al. [21] introduced search schemes, which optimize the search process by partitioning P into multiple parts and gradually allowing for more errors as matching progresses. A search scheme is a collection of searches that reduces the search space by imposing specific error bounds across pattern segments, efficiently covering every possible error distribution. For example, the search scheme based on the pigeonhole principle divides P into K+1 parts, with each search ensuring that at least one part matches exactly [24]. More advanced search schemes for higher error thresholds have been proposed [25, 26], providing a foundation for improved lossless approximate pattern matching tools such as Columba [27, 28, 29, 26].

It is critical that a bidirectional full-text index is used to support search schemes, as they require the ability to match a pattern P in both directions, starting at any position within P and switching direction as needed. The demonstrated efficiency of search schemes, such as in the lossless read-mapper Columba, motivates the need for a fast bidirectional full-text index in run-length compressed space.

### 2.2 Character extensions in various indexes

#### 2.2.1 Finding exact occurrences with the FM-index and r-index

The uncompressed FM-index supports counting functionality in O(n) space and O(m) time, where m is the length of pattern P [4]. Consider the interval [s,e] in the FM-index corresponding to all sorted suffixes of T prefixed by pattern P. The interval $\left[s^{\prime },e^{\prime }\right]$ for P’s extension cP can be found as $s^{\prime }=\text{C}[c]+{\text{rank}}_{c}(\text{BWT},s)$ and $e^{\prime }=\text{C}[c]+{\text{rank}}_{c}(\text{BWT},e+1)-1$. Here, C[c] denotes the number of characters in T strictly smaller than c, and ${\text{rank}}_{c}(\text{BWT},i)$ counts the occurrences of character c in the BWT before index i. These operations can be executed in constant time if the BWT is represented as a collection of |Σ| bitvectors with constant-time rank support. For readers less familiar with counting functionality using backward search in the FM-index, a more extensive overview is provided in [30].

To address the FM-index’s space inefficiency, which increases linearly with the size of the search text, Gagie et al. [10, 11] introduced the r-index. The r-index offers counting functionality in O(r) space with a time complexity of $O\left(m\cdot \log\left(\log_{w}(|\text{Σ}|+n/r)\right)\right)$, where w=Ω(log(n)) is the machine word size. Although the counting process in the r-index is conceptually similar to that in the FM-index, it requires a more complex combination of operations. Specifically, each character extension involves a combination of access, rank, select, and conditional operations performed on multiple data structures, which, collectively, cannot be performed in constant time. For further details, refer to [11]. This complexity leads to more random access operations and, consequently, cache misses.

#### 2.2.2 Constant-time LF operations with the move structure

The LF(i) operation maps the character at index i in the BWT or L (the last column of the lexicographically ordered rotations of T) to its corresponding character in F (the first column of the lexicographically ordered rotations of T). Nishimoto and Tabei [16] introduced the move structure as an alternative to the r-index that achieves LF operations in constant time. Their key insight is that LF mappings within a single run map to consecutive positions in F. Instead of mapping a single position in L to F, *input intervals* in L are mapped to *output intervals* in F. For example, in the index illustrated in Table 1, the input interval [2,5] from the second BWT run corresponds to the output interval [11,14] in F.

Conceptually, the move structure contains an LF move table with r rows: one for each run in the BWT. Following the notation from Zakeri et al. [17], each row contains four elements: the character c of the run, the starting index p of its input interval, the starting index π=LF(p) of its output interval, and the run index ξ that contains π. The move table $M_{\text{LF}}$ for the example of Table 1 is shown in Table 2. Note that ξ is not injective: multiple input runs can have their π residing in the same output run.

To perform the LF operation on a given BWT index i, we need the run index j that contains i to access move table $M_{\text{LF}}$. Assuming we know both i and j and aim to compute LF(i), Algorithm 1 outlines the process. We start by determining the offset between i and the start of its run. With this offset and the start of the output interval for run j, we compute LF(i). For potential subsequent LF operations, we must also find the run index containing LF(i) using fast forwarding functionality. Fast forwarding is necessary when run $M_{\text{LF}}[j].\xi$ does not contain LF(i). For example, to find the run index corresponding to LF(5)=14, where i=5 resides within BWT run j=1, we observe that run index $M_{\text{LF}}[1]\cdot \xi =6$ (covering the interval [11, 11]) does not include BWT index 14. In such cases, we traverse subsequent runs until the correct run is located using the fastForward function. We ensure that the access on line 2 is never out-of-bounds by adding an extra row at the bottom of the move table $M_{\text{LF}}$, such that $M_{\text{LF}}[r]\cdot p=n$. For example, in Table 2, $M_{\text{LF}}[12]\cdot p=19$.

The LF operation, as described in Algorithm 1, is more cache-friendly than the alternative in the r-index. It involves jumping to row $M_{\text{LF}}\left[M_{\text{LF}}[j]\cdot \xi \right]$ and possibly accessing subsequent rows. Fetching these subsequent rows translates to linear memory access (streaming) and can be efficiently performed in contemporary computer architectures. For true constant-time LF operations, the number of steps in the fast forwarding function should be limited. While Nishimoto and Tabei [16] suggested balancing or splitting the input and output intervals to achieve this, Zakeri et al. [17] found that splitting these intervals did not result in a notable speedup when implementing character extensions in Movi, nor did we (see further). Therefore, we maintain the original runs in this paper. Technically, this leads to an O(r) worst-case time complexity. In practice, however, this is the more efficient choice for building and storing the index and does not noticeably impact search performance.

**Algorithm 1:** Find $\text{LF}(i)=M_{\text{LF·moveStep}}(i,j)$ given tuple (i,j), where j is the run index that contains BWT index i.

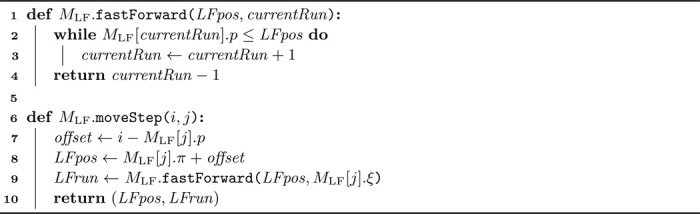

#### 2.2.3 Finding approximate occurrences with the bidirectional FM-index and br-index

The bidirectional FM-index [24] can extend a pattern P to Pc or cP by maintaining synchronized intervals over SA and ${\text{SA}}^{\text{rev}}$. Here, ${\text{SA}}^{\text{rev}}$ is the suffix array of $T^{\text{rev}}$ (the reverse of T). To enable bidirectional extension, certain components corresponding to the FM-index of $T^{\text{rev}}$ are stored; see [30] for details. Consider intervals [s,e] and $\left[s^{\text{rev}},e^{\text{rev}}\right]$ for P in SA and $P^{\text{rev}}$ in ${\text{SA}}^{\text{rev}}$. To extend P to cP, we find $\left[s^{\prime },e^{\prime }\right]$ for cP in SA as in Section 2.2.1. Updating $\left[s^{\text{rev}},e^{\text{rev}}\right]$ involves recognizing that $\left[s^{\text{rev}\prime },e^{\text{rev}\prime }\right]\subseteq \left[s^{\text{rev}},e^{\text{rev}}\right]$ due to $P^{\text{rev}}c$ being prefixed by $P^{\text{rev}}$. Moreover, all suffixes in ${\text{SA}}^{\text{rev}}$ prefixed by $P^{\text{rev}}a$, a≺c, are sorted before those within the interval $\left[s^{\text{rev}\prime },e^{\text{rev}\prime }\right]$. If we compute the cumulative number of occurrences x of aP in T, for all characters a≺c, using the procedure from Section 2.2.1, interval $\left[s^{\text{rev}\prime },e^{\text{rev}\prime }\right]$ in ${\text{SA}}^{\text{rev}}$ can be found as $e^{\text{rev}\prime }=s^{\text{rev}}+x$ and $e^{\text{rev}\prime }=s^{\text{rev}}+x+y-1$, where $y=e^{\prime }-s^{\prime }+1$ is the count of cP occurrences in T. Extending P to Pc is done symmetrically.

Arakawa et al. [20] introduced the br-index, an extension of the r-index taking up $O\left(r+r^{\text{rev}}\right)$ space, $r^{\text{rev}}$ being the number of runs in the BWT of $T^{\text{rev}}$. Interval synchronization in the br-index parallels the method above but uses more intricate combinations of access, rank, select, and conditional operations on various data structures. Similar to the r-index, this leads to multiple cache misses and hence, slower performance. Additionally, the problem exacerbates as the number of occurrences for all a≺c must be found, requiring O(|Σ|) rank operations on BWT or ${\text{BWT}}_{\text{rev}}$.

#### 2.2.4 The toehold lemma

Once all required (bidirectional) character extensions have been performed and the corresponding occurrence interval(s) have been found in the index, the user may want to locate these occurrences in the original search text. To enable this post-search locating process, a toehold update must follow each character extension [10, 11]. The toehold is an SA value that corresponds to one of the occurrences within the current SA interval, maintained throughout the search process. In bidirectional pattern matching, this toehold does not always correspond to the start or end of the SA interval, unlike in exact, backward pattern matching (as seen in the r-index), where the toehold systematically corresponds to the last entry in the SA interval.

The toehold is updated after each character extension through a combination of core operations, similar to the LF operation. Our approach to the toehold update closely follows that of the br-index; for a more detailed explanation, see [20]. Note that Arakawa et al. refer to the toehold as the SA sample.

### 2.3 Locating occurrences in various indexes

#### 2.3.1 From one toehold to all occurrences in the r-index

Locating the coordinates of all occurrences in the search text for a given SA interval begins with the toehold, which corresponds to the SA value of one of the entries in this final SA interval. In the case of the r-index, which performs exact backward matching, the toehold specifically represents the last entry of the SA interval [10, 11]. Using this toehold, we must find the SA values of the preceding entries within the SA interval. This can be achieved using the ϕ operation, defined as follows (where ISA is the inverse of the SA):

$$\phi (i)=\{\begin{array}{ll} \text{SA}[\text{ISA}[i]-1] & \text{if}\;\text{ISA}[i]>0\\ \text{SA}[n-1] & \text{if}\;\text{ISA}[i]=0 \end{array}$$

For an SA interval of width m (indicating m occurrences), the ϕ operation thus needs to be applied m−1 times, starting with the toehold, to locate all occurrences.

In the r-index, the ϕ operation is implemented in O(r) space with a time complexity of $O\left(\log\left(\log_{w}(n/r)\right)\right)$, where w=Ω(log(n)) is the machine word size. Similar to the LF operation and character extensions in the r-index (see Section 2.2.1), performing the ϕ operation involves a complex combination of access, rank, select, and conditional operations across multiple data structures, which collectively do not allow for constant-time execution. Moreover, this complexity results in unpredictable random access patterns and, consequently, more cache misses. For further details on the ϕ operation, refer to [11].

#### 2.3.2 Constant-time ϕ operations with the move structure

Similar to how a move table supports the LF operation in constant time in Nishimoto and Tabei’s move structure (see Section 2.2.2), a move table can also be constructed for constant-time ϕ operations. This approach is based on the observation that also the ϕ operation comprises r input intervals, each of which consecutively maps to its corresponding output interval. However, while the consecutive mapping is obvious for LF, it is less intuitive for ϕ.

Table 1 shows the ϕ operation and its corresponding input intervals. The correctness of the ϕ column can be validated using the SA column. We observe that ϕ input intervals are defined such that a new input interval begins at index k if the character T[k] marks the start of a run in the BWT. In other words, if k is the start of a ϕ input interval and k=SA[i], then i indicates the start of a BWT run. For example, when i=9, which signifies the start of a BWT run, then k=SA[i]=4 correctly marks the beginning of a ϕ input interval. Observe in Table 1 that ϕ indeed maintains consecutiveness within the ϕ input intervals. The formal proof that this property always holds is detailed in [11].

Once the input and output intervals are established, the move table $M_{\phi }$ can be built similarly to $M_{\text{LF}}$ in Section 2.2.2, but without the c column. The move table $M_{\phi }$, corresponding to the example in Table 1, is shown in Table 3 on the left. Additionally, the ϕ operation can be executed analogously to the LF operation (see Algorithm 1): $\phi (i)=M_{\phi }\cdot \text{moveStep}(i,j)$, where j denotes the ϕ run containing index i.

Note that the number of steps in the fast forwarding process should be limited to maintain constant-time ϕ operations. Unlike for the LF operation, balancing the input and output intervals for $M_{\phi }$ will significantly impact practical performance in this context. This is due to a key difference in how BWT and ϕ runs behave. In a pan-genome, we expect all BWT run sizes to be relatively similar, as homologous regions tend to group together. However, for ϕ runs, there is no clear expectation about their sizes. In practice, we often observe that the distribution of ϕ run sizes is heavily right-tailed. For this reason, the move table $M_{\phi }$ will be balanced in the context of this paper. The balanced version of the move table on the left in Table 3, is shown on the right. The balancing algorithm is discussed in [16]. Note that balancing a move table never increases the number of rows by more than a factor of two, ensuring that the theoretical space and time complexities remain valid.

In summary, using the move table allows the ϕ operation to achieve an O(1) time complexity and, more importantly, operate efficiently due to the high cache locality, as explained in Section 2.2.2.

#### 2.3.3 From one toehold to all occurrences in the br-index

As mentioned in Section 2.2.4, the toehold does not necessarily represent either the start or the end of the SA interval after bidirectional pattern matching. Consequently, it is necessary to retrieve not only the preceding entries in the SA interval but also the succeeding ones. To obtain the latter, we use the inverse of the ϕ operation:

$$\phi^{-1}(i)=\{\begin{array}{ll} \text{SA}[\text{ISA}[i]+1] & \text{if}\;\text{ISA}[i]<n-1\\ \text{SA}[0] & \text{if}\;\text{ISA}[i]=n-1 \end{array}$$

Analogous to the ϕ operation, $\phi^{-1}$ can be performed in $O\left(\log\left(\log_{w}(n/r)\right)\right)$ time in the br-index (where w=Ω(log(n)) is the machine word size), but in practice, its complex memory access pattern leads to multiple cache misses.

The remaining challenge is that the number of preceding and succeeding occurrences in the interval is not known a priori. In other words, while we know m−1 (with m being the width of the interval) ϕ or $\phi^{-1}$ operations must be performed, the exact split between these operations is unknown. To address this, Arakawa et al. utilize the permuted longest common prefix array (PLCP), where PLCP[i]=LCP[ISA[i]] [20]. They observed that for an SA interval [b,e] representing a pattern P, we have PLCP[SA[b]] <|P|,PLCP[SA[e+1]]<|P|, and PLCP[SA[i]]≥|P| for b<i≤e. Thus, accessing the PLCP array after each ϕ or $\phi^{-1}$ operation determines when to stop locating occurrences. As in Arakawa et al.’s implementation, we store the PLCP using a predecessor data structure, which takes O(r) memory and requires $O\left(\log\left(\log_{w}(n/r)\right)\right)$ time per access, where w=Ω(log(n)) is the machine word size. Refer to [20] for further details.

## 3 Bidirectional move structure

While Nishimoto and Tabei’s move structure with constant-time LF and ϕ support shows promise, practical implementations remain scarce. Brown et al. [31] analyzed time-space tradeoffs and proposed a compressed implementation of the move table that efficiently counts exact occurrences while using less space. Zakeri et al. [17] introduced Movi, a fast and cache-efficient full-text pan-genome index that supports computing matching statistics and pseudo-matching lengths, useful for finding maximal exact matches. Bertram et al. [18] proposed Move-r, a highly optimized version of the r-index supporting (exact) count and locate queries. However, to our knowledge, no practical bidirectional move

**Algorithm 2:** Let [s,e] be the interval over SA corresponding to P, which we want to extend to cP. $R_{s}$ and $R_{e}$ are the runs containing s and e, respectively. Functions walkToNextRun and walkToPreviousRun return the SA indices $s_{c}$ and $e_{c}$ (along with their run indices $R_{s,c}$ and $R_{e,c}$) which indicate the smallest subinterval within [s,e] containing all occurrences of c in the BWT.

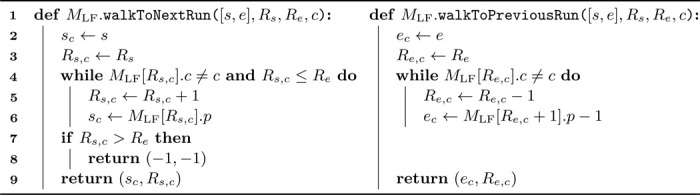

**Algorithm 3:** Extend pattern P, represented by [s,e], corresponding to respectively run index $R_{s}$ and $R_{e}$, to cP. The algorithm returns the updated interval $\left[s^{\prime },e^{\prime }\right]$, corresponding to respectively run index $R_{s}^{\prime }$ and $R_{e}^{\prime }$, for cP.

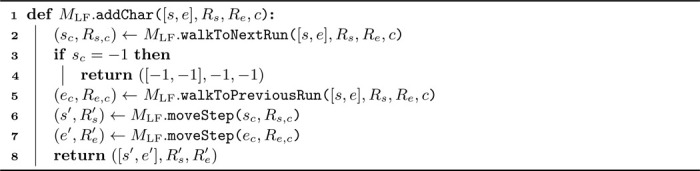

structure exists that supports finding approximate occurrences of a pattern in a search text. In this section, we introduce our bidirectional move structure, and detail how it (cache-)efficiently performs bidirectional character extensions and ϕ or $\phi^{-1}$ operations using $O\left(r+r^{\text{rev}}\right)$ space.

### 3.1 Bidirectional character extensions

#### 3.1.1 Backward search

We elaborate on unidirectional search based on the LF functionality discussed in Section 2.2.2. As for the LF operation, run indices corresponding to the current SA interval boundaries are required for character extension. Consider interval [s,e] in SA for pattern P, with $R_{s}$ and $R_{e}$ the runs containing s and e. To extend P to cP, the LF functionality cannot be applied directly to the interval boundaries, as runs $R_{s}$ and $R_{e}$ may not match c. Instead, LF must be performed on subinterval $\left[s_{c},e_{c}\right]\subseteq [s,e]$, which is the smallest interval containing all occurrences of c in the BWT in [s,e]. We again require run indices $R_{s,c}$ and $R_{e,c}$ for $s_{c}$ and $e_{c}$. To find $R_{s,c}$ and $R_{e,c}$ using only the move tables, we simply walk down and up along the rows until finding an instance of c, inspired by Zakeri et al.’s repositioning in Movi-default [17]. Function walkToNextRun in Algorithm 2 demonstrates this approach. If no occurrences of c exist within [s,e] (checked on line 7), −1 is returned for both values.

Function walkToPreviousRun in Algorithm 2 is similar to function walkToNextRun, but the check on line 7 is no longer necessary as the function is always executed in circumstances where it is known that c occurs in the BWT within [s,e] (usually by running walkToNextRun first). Also, on line 6 in function walkToPreviousRun, a subsequent row is accessed since the end indices of the runs are not stored in the move table. Note that this access can never be out-of-bounds. Function walkToPreviousRun can also be used to update the toehold if required for locating, details are omitted here.

Algorithm 3 then combines the walking from Algorithm 2 and the LF from Algorithm 1 into the functionality to extend a pattern P to cP. Algorithm 2 and 3 have a worst-case time complexity of O(r) but are very fast in practice. We also explored constant-time alternatives for Algorithm 2, such as storing additional bitvectors representing the run heads, supporting rank and select operations. However, this option performed worse in practice, both in memory usage and speed, due to complex memory access patterns.

#### 3.1.2 Bidirectional search

Similar to the bidirectional FM-index and r-index, we incorporate an additional component to represent the reverse search text $T^{\text{rev}}$ for bidirectional search with our bidirectional move structure. Specifically, we store the move table $M_{\text{LF}}^{\text{rev}}$ corresponding to $T^{\text{rev}}$. Table 4 shows $M_{\text{LF}}^{\text{rev}}$ corresponding to $M_{\text{LF}}$ in Table 2. For completeness, the FM-index corresponding to $T^{\text{rev}}$ is shown in Table S1 in the additional file.

Using the combination of $M_{\text{LF}}$ and $M_{\text{LF}}^{\text{rev}}$, we can synchronize the intervals corresponding to a search pattern P over SA and $P^{\text{rev}}$ over ${\text{SA}}^{\text{rev}}$. Algorithm 4 details how pattern P can be extended to cP while keeping both intervals up to date. The approach is conceptually similar to that described in Section 2.2.3. Extending P to the right, i.e., to $P_{c}$, is analogous and is detailed in supplementary Algorithm S1 in the additional file.

##### Memoization.

For clarity, the algorithms were built step-by-step. Thus, the time complexity for Algorithm 4 and S1 is O(|Σ|⋅r) and $O\left(|\text{Σ}|\cdot r^{\text{rev}}\right)$, respectively, as all characters a≺c must be checked (line 8). Alternatively, combining Algorithms 1 to 4 can achieve O(r) bidirectional character extensions instead of O(|Σ|⋅r). As |Σ| is small in the context of this paper, this is mostly a theoretical improvement. To achieve this, consider the calls to function addChar in the for-loop on line 8 (and on line 2) of Algorithm 4, causing the |Σ| factor. As Algorithm 3 is called |Σ| times, the following O(r) time functions are also executed |Σ| times:

- walkToNextRun (Alg. 2)
- walkToPreviousRun (Alg. 2)
- moveStep (the LF operation, Alg. 1), twice

For walkToNextRun (i), the while-loop on line 4 in Algorithm 2 could be merged with the for-loop on line 8 of Algorithm 4 to continue walking until the first occurrence of each character a≺c is found. This results in one O(r) walk, keeping each $\left(s_{a},R_{s,a}\right)$ tuple in memory. Similarly, walkToPreviousRun (ii) can be reduced to one O(r) walk, keeping

**Algorithm 4:** Update intervals $\left([s,e],R_{s},R_{e}\right)$ and $\left(\left[s^{\text{rev}},e^{\text{rev}}\right],R_{s}^{\text{rev}},R_{e}^{\text{rev}}\right)$ corresponding to P, to intervals $\left(\left[s^{\prime },e^{\prime }\right],R_{s}^{\prime },R_{e}^{\prime }\right)$ and $\left(\left[s^{\text{rev}\prime },e^{\text{rev}\prime }\right],R_{s}^{\text{rev}},R_{e}^{\text{rev}}\right)$ that correspond to cP.

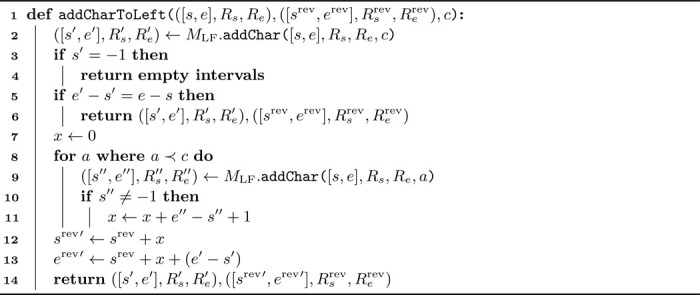

**Algorithm 5:** Update run indices using binary search to find the correct run containing index i. The algorithm uses the move table $M_{\text{LF}}$ upon which it is called.

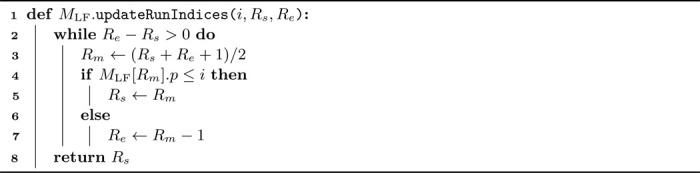

each $\left(e_{a},R_{e,a}\right)$ tuple in memory.

Then, on each $\left(s_{a},R_{s,a}\right)$ and $\left(e_{a},R_{e,a}\right)$ tuple, moveStep (iii) is performed, which is O(r) due to the fastForward procedure. However, fast forwarding is always limited to the output interval corresponding to the input run/interval. Since the input runs for different characters a≺c are distinct, fast forwarding occurs in disjoint output intervals, collectively summing to O(r) steps. If $R_{s,a}=R_{e,a}$ (i.e., they have the same output interval), redundant fast forwarding can be avoided through memoization as well.

As such, the total combination of walking steps and fast forwarding steps necessary to perform bidirectional character extensions sums to O(r) instead of O(|Σ|⋅r). Analogously, Algorithm S1 can be performed in $O\left(r^{\text{rev}}\right)$ time. We emphasize that in practice, the number of walking/fast forwarding steps is small, and due to linear memory access (streaming), the bidirectional character extensions are very fast.

#### 3.1.3 Direction switches

In Algorithm 4, run indices $R_{s}^{\text{rev}}$ and $R_{e}^{\text{rev}}$ are not updated, and the same holds for $R_{s}$ and $R_{e}$ in Algorithm S1. Updating these indices at each synchronization step is not efficient nor necessary; they are required only when switching search direction.

In practice, patterns can be split into multiple parts using different partitioning techniques (e.g., dynamic partitioning [27]), see Section 2.1. For the sake of the example, however, consider pattern P=“TATGTTGGT”, split into two parts: “TATGT|TGGT”, for which we match the first part to the left starting at the separation point. After five calls to addCharToLeft, the state is $\left([s,e],R_{s},R_{e}\right)$, $\left(\left[s^{\text{rev}},e^{\text{rev}}\right],R_{s}^{\text{rev}},R_{e}^{\text{rev}}\right)=$ ([11,12],6,7), ([16,17],0,12). Indeed, neither ${\text{SA}}^{\text{rev}}$ index 16 nor 17 lies within their corresponding runs of 0 and 12. To switch direction and extend the match to the right, we first update $R_{s}^{\text{rev}}$ and $R_{e}^{\text{rev}}$.

We employ a binary search over the move rows, starting with the current (outdated) run indices, detailed in Algorithm 5. For our example,$M_{\text{LF}}^{\text{rev}}\cdot \text{updateRunIndices}(16,0,12)=10$ and $M_{\text{LF}}^{\text{rev}}\cdot \text{updateRunIndices}(17,0,12)=11$. With the correct run indices, we can extend the match to the right.

Algorithm 5 has a time complexity of O(log(r)). Note that in practice, direction switches are infrequent. Moreover, the binary search often operates with narrow starting intervals. In the example above, we started from the complete interval over all move rows. For another switch back to the left, we would call $M_{\text{LF}}\cdot \text{updateRunIndices}(i,6,7)$.

### 3.2 ϕ and $\phi^{-1}$ operations

Analogous to the ϕ move table $M_{\phi }$ discussed in Section 2.3.2, which allows for cache-efficient, constant-time ϕ operations, we can introduce a second move table, $M_{\phi^{-1}}$, to similarly support constant-time $\phi^{-1}$ operations. Table 1 shows the $\phi^{-1}$ operation and its corresponding input intervals. Table 5 presents the corresponding $M_{\phi^{-1}}$ table, showing the unbalanced version on the left and the balanced version on the right.

The approach of replacing the original ϕ and $\phi^{-1}$ operations of the (b)r-index with those based on move tables is conceptually similar to the replacement of LF operations discussed in Sections 2.2.2 and 3.1.2. However, a key distinction arises: the toehold computations (see Section 2.2.4) still depend on a subset of the data structures designed for the original ϕ and $\phi^{-1}$ operations in the (b)r-index. Thus, while the original LF data structures can be entirely replaced by move tables, a portion of the original ϕ and $\phi^{-1}$ operation data structures must be retained in this case.

Consequently, choosing whether to implement ϕ and $\phi^{-1}$ operations with move tables requires careful consideration of the trade-off between increased RAM usage for faster execution (with ϕ move tables) and reduced RAM usage at the expense of lower performance (without ϕ move tables).

### 3.3 Bit-packing the move tables

Given that the move tables are crucial for supporting the core operations of pattern matching, it is imperative that they are implemented efficiently in terms of both space and performance. First and foremost, the tables should be stored in row-major order, as all elements within a row are required to perform the LF, ϕ, or $\phi^{-1}$ operations.

To store the actual elements within each row, there are two primary options. The first and most straightforward option is to store every integer using a fixed number of bits, independent of the pan-genome size *n* and the number of rows in the move table. Given that n can be quite large, we generally use 64-bit integers. We refer to this option as full move tables for the remainder of this paper. While this approach provides a fast solution in terms of runtime, it results in a significant number of unused bits. Specifically, the p and π columns require at most $\left\lceil \log_{2}(n)\right\rceil$ bits, while the ξ column needs at most $\log_{2}(\text{\# rows})$ bits. The number of rows varies by move table: r+1 for $M_{\text{LF}}$, $r^{\text{rev}}+1$ for $M_{\text{LF}}^{\text{rev}}$, and $r^{\prime }\geq r+1$ for $M_{\phi }$ and $M_{\phi^{-1}}$, depending on the number of balanced input and output intervals.

The second option is to store each element using its dedicated number of bits. Consequently, one row will occupy at least $\text{bitsPerRow}=2\cdot \log_{2}(n)+\log_{2}(\text{\# rows})$ bits. For LF rows, an additional 3 bits must be added to store the BWT run character. To ensure that a row always starts on a new byte (which enhances cache performance), we calculate the number of bytes per row as $\text{bytesPerRow}=\lceil \frac{\text{bitsPerRow}}{8}\rceil$. The move table can then be stored as a single large data block, with all rows stored sequentially. Accessing a specific row *j* requires retrieving the bytes in the range [bytesPerRow·j,bytesPerRow·(j+1)−1]. Finally, with bit-shifting and masking operations, we can extract the specific elements from the row bytes. We refer to this option as bit-packed move tables for the remainder of this paper.

## 4 Results

### 4.1 Data and hardware

We built pan-genomes from two datasets^1^: i) 512 human chromosome 19 haplotypes from the 1000 Genomes Project [32], and ii) 3264 *Escherichia coli* strains downloaded from NCBI’s Reference Sequence (RefSeq) collection. Characters ‘N’ were removed from these genomes. The chromosomes or strains are concatenated into one string, from which the indexes are built. Table 6 provides more detailed statistics regarding the pan-genomes we used for benchmarking. Note that the last column with the ratio between n and r confirms that the human pan-genome is more repetitive and conserved than the bacterial pan-genome.

The index construction process for b-move supports two options: in-memory or prefix-free parsing based [33]. The in-memory method is fast but requires a substantial amount of RAM for large pan-genomes. The prefix-free parsing based method is slower but uses much less memory. For example, constructing the index (including balancing the ϕ tables) for the largest pan-genome of 512 human chromosome 19 haplotypes takes 2 hours and 520 GB in-memory, whereas prefix-free parsing based takes 7 hours and 84 GB, suitable for most workstations.

For benchmarking the human pan-genomes, we consider 100000 Illumina HiSeq 2500 reads (151 bp) randomly sampled from a larger whole genome sequencing dataset (accession number SRR17981962). For the bacterial pan-genomes, we use 100000 Illumina NovaSeq 6000 reads (151 bp) randomly sampled from a larger whole genome sequencing dataset (accession number SRR28249370). Benchmarks were performed on a Red Hat Enterprise Linux 8 system, using a single core of two 18-core Intel^®^ Xeon^®^ Gold 6140 CPUs running at a base clock frequency of 2.30GHz with 177 GiB of RAM. To account for variability in runtime, all reported runtimes are based on the median of 10 runs. They exclude the time to read the index from and write the output to disk.

### 4.2 Character extension performance

#### 4.2.1 Analysis of the number of walking and fast forwarding steps

**Figure 1:**
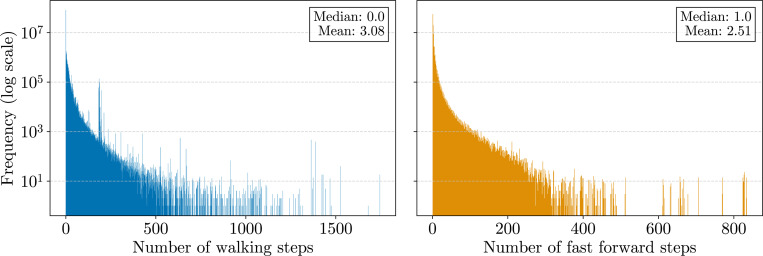
Log scale histograms for the number of walking and fast forwarding steps required per successful bidirectional character extension (96363328 in total) for finding all SA intervals corresponding to all occurrences up to 3 mismatches. We aligned 100000 Illumina reads of length 151 bp and their reverse complements to the pan-genome composed of 512 human chromosome 19 haplotypes.

We analyzed the O(r) and $O\left(r^{\text{rev}}\right)$ operations in each bidirectional character extension (see Section 3.1.2). We conducted an experiment to identify all SA intervals (i.e., without locating) corresponding to all occurrences with up to 3 mismatches for 100000 reads in the pan-genome of 512 human chromosome 19 haplotypes. Figure 1 shows the number of walking and fast forwarding steps for each successful bidirectional character extension in this experiment. Most extensions require very few steps (note the log scale): the mean number of walking steps per extension is 3.08, and the mean number of fast forwarding steps per extension is 2.51 (with at least one fast forwarding step required for each LF operation, see Alg. 1).

A similar analysis for the *E. coli* pan-genome of 3264 strains yields comparable results: a mean of 0.52 walking steps per extension and a mean of 1.94 fast forwarding steps per extension (not shown). Thus, these worst-case time complexities of O(r) and $O\left(r^{\text{rev}}\right)$ have minimal impact on the actual performance of character extensions.

#### 4.2.2 Character extension runtimes

**Figure 2:**
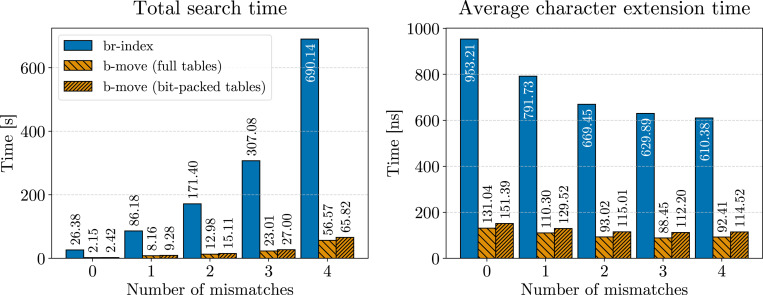
Benchmark results for finding all SA intervals corresponding to all occurrences up to a certain number of mismatches (x-axis). The left panel displays the total search time, while the right panel shows the average execution time for the core bidirectional character extension functionality. We aligned 100000 Illumina reads of length 151 bp and their reverse complements to the pan-genome composed of 512 human chromosome 19 haplotypes.

In Figure 2, we compare the efficiency of bidirectional character extensions using the original br-index implementation^2^ by Arakawa et al. [20], and our bidirectional move structure, referred to as b-move, using both full and bit-packed LF move tables (see Section 3.3). The left panel displays the total time for finding all SA intervals for occurrences up to a specific number of mismatches (i.e., no locating). To ensure a fair comparison, we adjusted b-move’s parameters to closely match the implementation of the br-index (e.g., Hamming distance, the use of the pigeonhole principle, and no further optimizations). As shown in the chart, both the full and bit-packed LF move tables enable b-move to out-perform the br-index by a factor of at least 9 to 11 in total search time. Note that while the RAM usage varies significantly between the full and bit-packed versions (2.6 GB and 1.1 GB, respectively), the bit-packed version incurs only a modest performance reduction of around 15%.

While we aimed for similar parameters, implementation differences beyond core character extension functionality may affect the total search time. For example, small differences in the search strategies can lead to a different number of character extensions performed to obtain the same result. To address this, we specifically benchmarked the character extension functionality described in Algorithms 4 and S1, along with their counterparts in the br-index. We used a built-in instruction (constant rdtscp) to count CPU cycles and scaled the average cycle count to time using the clock frequency. Given that cache misses consume dozens of CPU cycles, the time per character extension also serves as a proxy for cache misses. The right panel of Figure 2 shows a speed-up of 5 to 7 × in favor of b-move. Moreover, we observe a similar performance difference between full and bit-packed versions as in the left panel. This trade-off offers substantial memory savings however, making the bit-packed version the most efficient choice in practice. In the remainder of the benchmarks, we opt for bit-packed LF move tables.

Also note that the time for a single bidirectional character extension in the br-index decreases when allowing more mismatches. This is because with more allowed paths in the search tree, certain memory accesses are repeated more frequently, either to determine the widths of all intervals for a≺c or to extend with a itself. Consequently, the number of cache misses also decreases somewhat at higher error rates. This effect is mitigated in b-move due to its superior cache efficiency.

### 4.3 Locating performance

#### 4.3.1 Analysis of the number of fast forwarding steps

**Figure 3:**
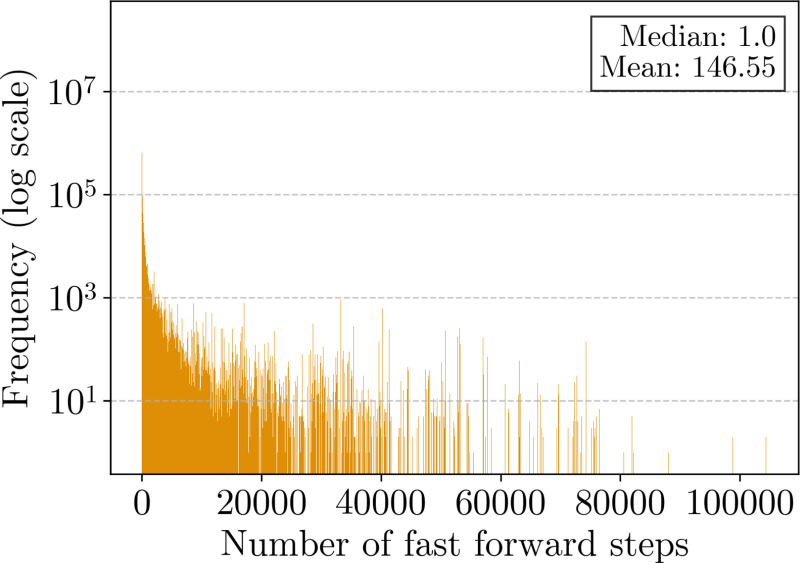
Log scale histogram for the number of fast forwarding steps required per ϕ or $\phi^{-1}$ operation (355041072 in total) for finding all occurrences up to 3 mismatches. We aligned 100000 Illumina reads of length 151 bp and their reverse complements to the pan-genome composed of 3264 *E. coli* strains, using *unbalanced* ϕ move tables. Using *balanced* ϕ move tables, the mean number of fast forwarding steps is reduced to 1.49 (not shown).

When implementing the ϕ and $\phi^{-1}$ operations using *unbalanced* move tables, the number of fast forwarding steps required (see Alg. 1) is O(r). To evaluate the practical impact of this, we identified all occurrences with up to 3 mismatches for 100000 reads in the pan-genome of 3264 *E. coli* strains. Figure 3 shows the number of fast forwarding steps required per ϕ or $\phi^{-1}$ operation during this process.

This analysis reveals significant differences compared to the analogous experiment for character extensions (see Section 4.2.1): the histogram for the ϕ and $\phi^{-1}$ operations shows a much more pronounced right tail compared to that for character extensions. As a result, the mean number of fast forwarding steps per ϕ or $\phi^{-1}$ operation is substantially higher, at 146.55 steps. Thus, unlike in character extensions, fast forwarding in ϕ and $\phi^{-1}$ operations has a notable impact on locating performance.

To mitigate this, we balance the ϕ and $\phi^{-1}$ move tables, as discussed in Section 2.3.2. When balanced, the maximum number of fast forwarding steps is capped at 4, with the mean number of steps reduced to 1.49. Therefore, for the remainder of this paper, we assume balanced ϕ and $\phi^{-1}$ move tables.

#### 4.3.2 Locating runtimes

**Figure 4:**
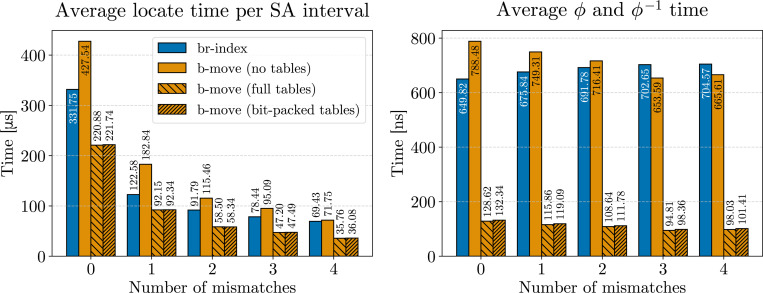
Benchmark results for finding all occurrences with up to a certain number of mismatches (x-axis). The left panel presents the average locate time per SA interval, while the right panel shows the average execution time for the core ϕ and $\phi^{-1}$ operations. The experiment aligns 100,000 Illumina reads of length 151 bp and their reverse complements to the pan-genome composed of 3264 *E. coli* strains.

Figure 4 provides an in-depth comparison of locating efficiency for the br-index and b-move, evaluating three configurations of b-move: no ϕ move tables (analogous to the br-index), full ϕ move tables, and bit-packed ϕ move tables. As in Section 4.2.2, b-move’s parameters were adjusted to closely match the br-index implementation.

The right panel again shows the average execution time for ϕ and $\phi^{-1}$ operations, measured using built-in CPU cycle count instructions. The performance of b-move’s original ϕ and $\phi^{-1}$ operations (without move tables) is comparable to that of the br-index, with minor variations likely due to differences in call patterns introduced by the approximate pattern matching approach in the implementation layer above, potentially leading to a distinct pattern of cache misses. Using move table-based ϕ and $\phi^{-1}$ operations yields a 5 to 7 × speedup over the original implementations.

The left panel of Figure 4 presents the average locate time required to map one SA interval to all corresponding occurrences in the pan-genome. As detailed in Section 2.3.3, this process involves repeated ϕ and $\phi^{-1}$ operations, accesses to the PLCP array, and overhead from creating new occurrence objects. Due to this overhead, the speedup from using move tables is less pronounced for the complete locating implementation, yet still maintains a 1.3 to 2 × improvement over the original approach. In scenarios where many occurrences must be located (e.g., in pan-genomes with numerous haplotypes or strains), this can significantly impact performance. The performance difference between the br-index and b-move’s implementation without ϕ move tables reflects a similar variability as was observed in the ϕ and $\phi^{-1}$ performance.

Note that there is again little difference in performance between full and bit-packed move tables for ϕ and $\phi^{-1}$ operations. Specifically, the runtimes are even closer together, likely due to the mean number of fast forwarding steps being somewhat lower than in Section 4.2. However, the full version requires 8.6 GB of memory for the ϕ move tables, while the bit-packed version reduces their memory usage to 4.3 GB. Thus, for the remainder of this paper, we focus on bit-packed ϕ and $\phi^{-1}$ move tables. Compared to the original locating functionality in b-move, the bit-packed tables still consume an additional 3.6 GB of space. If memory is a priority and the user can tolerate slower locating performance, the original functionality might be preferred, offering roughly half the speed but lower RAM usage. To accommodate this, we provide both options, though the move table-based implementation is the default.

Finally, note that the average locate time per SA interval decreases as the number of mismatches increases for all implementations. As more errors are allowed, the additional occurrences found are typically more specific to certain haplotypes or strains, resulting in fewer ϕ and $\phi^{-1}$ operations per locate.

### 4.4 Complete approximate pattern matching performance

Combining its efficient bidirectional character extensions and locating functionality, b-move becomes a practical tool for lossless approximate pattern matching of reads to large pan-genomes. As such, b-move can report *all* occurrences within a pre-specified Hamming or edit distance, in the form of one SAM line per occurrence. This functionality is similar to that provided by lossless read-mapper Columba (developed in the same research group), but Columba’s index is based on the bidirectional FM-index (O(n) memory requirements). b-move does not support reporting CIGAR strings, however, since the original text would have to be stored in memory for that.

**Figure 5:**
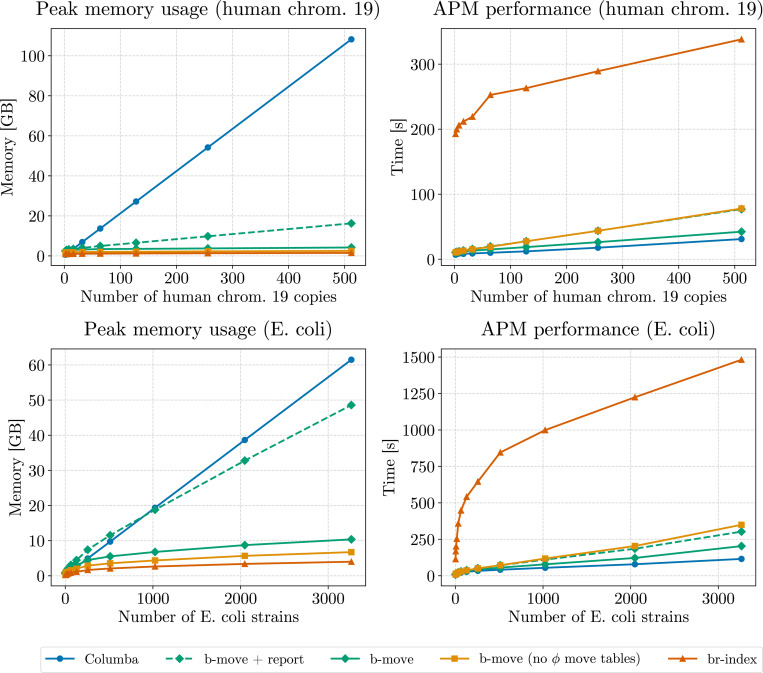
Benchmark results for approximate pattern matching using the br-index, b-move (with and without ϕ move tables), and Columba (suffix array sparseness of 8). Additionally, we include results for b-move (with ϕ move tables) with reporting functionality, which is not included in the other results. We aligned 100000 Illumina reads of length 151 bp and their reverse complements to pan-genomes for both human chromosome 19 and *E. coli*, across multiple sizes. We allowed for a maximum error distance of 3 (Hamming for br-index, edit for b-move and Columba).

To ensure practical efficiency in b-move, we incorporated several optimizations originally developed for Columba: optimized edit distance to reduce redundancy, superior search schemes replacing pigeonhole methods, a lookup-table to bypass matching the first 10-mers, dynamic pattern partitioning, and bit-parallel pattern matching. In Figure 5, we compare b-move with these optimizations in place (with and without ϕ move tables) with the br-index and Columba^3^ in terms of peak memory usage (left panels) and approximate pattern matching performance (right panels) across various pan-genome sizes. This performance evaluation includes both the human chromosome 19 pan-genomes (top panels) and the *E. coli* pan-genomes (bottom panels). For a fair comparison, note that optimizations in Columba requiring the original text are disabled.

Despite the fact that the *E. coli* pan-genomes are less repetitive than the human pan-genomes, both datasets demonstrate that the sublinear memory scaling characteristics of the br-index and b-move result in substantial memory reductions. Even for the largest pan-genomes, the br-index and both b-move options produce indexes that can be stored in the RAM of a regular laptop. The differences in size between the br-index, b-move without ϕ move tables, and b-move with ϕ move tables (which are more pronounced for *E. coli*) are put into perspective by the significant reduction relative to the FM-index-based tool. Regarding approximate pattern matching performance, b-move’s speed is of the same order of magnitude as Columba’s, albeit a constant factor of 1.3 to 1.8 (with ϕ move tables) or 2.5 to 3 (without ϕ move tables) larger. Conversely, the br-index is roughly 4 and 8 times slower than b-move without and with ϕ move tables, respectively.

In summary, b-move’s memory usage is orders of magnitude smaller than Columba’s for large, repetitive datasets, and closely resembles that of the br-index. Conversely, b-move outperforms the br-index by almost one order of magnitude while closely matching Columba’s performance in approximate pattern matching. Thus, we believe that b-move is the optimal index for scaling lossless approximate pattern matching to large pan-genomes.

#### Reporting the occurrences.

The charts in Figure 5 include a fifth line: b-move (with ϕ move tables) with reporting functionality. These results capture the overhead incurred by converting all found occurrences into SAM lines and maintaining them in memory throughout the search. Comparison between b-move + report and b-move alone highlights the substantial performance overhead of this reporting functionality. As a result, we excluded it from the main comparison. Nonetheless, an implementation for reporting is available both in b-move and Columba for users who require this feature. Note that the memory overhead due to reporting can be decreased significantly by writing out the alignments gradually throughout matching instead of buffering occurrences in memory (e.g., for *E. coli* the alignments comprise almost the same amount of space as Columba’s index). This can be achieved by implementing a dedicated reader and writer thread. The performance overhead, however, is less straightforward to address. One option would be to explore different techniques for extracting the desired information without relying on locating, such as tagging the index with additional metadata [19]. Such alternatives would avoid the locating and reporting process altogether.

#### Log-scale comparison.

**Figure 6:**
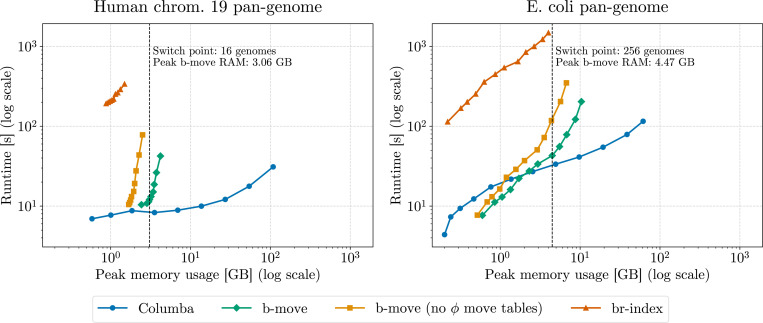
Log-scale time-memory comparison for approximate pattern matching using the br-index, b-move (with and without ϕ move tables), and Columba (suffix array sparseness of 8). We show the switch point for each pan-genome, which marks the minimum pan-genome size at which the b-move index becomes smaller than Columba. We aligned 100000 Illumina reads of length 151 bp and their reverse complements to pan-genomes for both human chromosome 19 and *E. coli*, across multiple sizes. We allowed for a maximum error distance of 3 (Hamming for br-index, edit for b-move and Columba).

Figure 6 presents a log-scale time-memory comparison chart, illustrating the same data as Figure 5. This visualization clarifies the relationships among the different tools and options, particularly for smaller pan-genomes as well. Clearly, while the br-index is compact, it consistently underperforms compared to the other methods.

In contrast, the comparison between Columba and b-move reveals a more pronounced trade-off. Figure 5 identifies the switch point for each pan-genome, which marks the minimum pan-genome size at which the b-move index becomes smaller than Columba. Below this switch point, Columba is preferable due to its smaller size and faster performance. However, beyond the switch point, Columba’s size increases rapidly, while b-move maintains comparable performance metrics with a significantly smaller index.

### 4.5 Index size

Though the (bidirectional) move structure offers faster approximate pattern matching than the (bidirectional) r-index with identical O(r) space complexity, its memory requirements are somewhat larger in practice. We evaluate this for the largest pan-genome (in terms of BWT runs) from Table 6: the pan-genome of 3264 *E. coli* strains. A detailed overview of the space usage of the different components can be found in Table S2 in the additional file.

For character extensions without locating functionality, the bidirectional move structure (3.1 GB) requires approximately 5.5 times more space than the bidirectional r-index (0.5 GB). To support locating functionality with ϕ move tables, the additional data structures in the bidirectional move structure (6.9 GB) require approximately double the space of the corresponding data structures in the bidirectional r-index (3.3 GB). Note that b-move supports both of these options at the choice of the end user.

In conclusion, b-move requires 10.0 GB of space with ϕ move tables and 6.4 GB of space without ϕ move tables, compared to 3.8 GB required for the bidirectional r-index. All of these indexes are manageable even on a standard laptop. In a broader context, this difference is reasonable given the substantial performance improvement offered by the move structure. This becomes even more evident when comparing the space usage with FM-index-based tools (as discussed in Section 4.4).

Note that the additional memory usage required to support toehold updates can still be decreased by applying the subsampling technique [34, 35].

## 5 Conclusion

We propose b-move, a cache-efficient, run-length compressed index supporting lossless approximate pattern matching against large pan-genomes. b-move can perform character extensions 5 to 7 times faster than the br-index and is narrowing the performance gap with non-compressed FM-index-based pattern matching. Similarly, b-move supports move table-based ϕ and $\phi^{-1}$ operations, also achieving speedups of up to 7 times with respect to the br-index. Additionally, b-move demonstrates favorable sublinear memory characteristics, being orders of magnitude smaller than the FM-index for large pan-genomes. For example, *all* 3264 available complete *E. coli* genomes on NCBI’s RefSeq collection can be compiled into a b-move index usable on a regular laptop. Future work includes integrating the PLCP in the ϕ and $\phi^{-1}$ move tables to reduce the number of cache misses even further. Moreover, we aim to optimize the reporting functionality to minimize both memory (by altering the writing system) and runtime (by researching alternatives such as tagging) overhead. We also aim to integrate subsampling functionality to further reduce memory usage of the index itself. Finally, we plan to further optimize the b-move construction process. The source code of b-move is written in C++ and is available at https://github.com/biointec/b-move under AGPL-3.0 license.

## Supplementary Material

Supplement 1

## Figures and Tables

**Table 1:** Example index for search text *T* = “CTATGTCATATGTTGGTC$” with ϕ, $\phi^{-1}$, and BWT run details.

i	T	ϕ	$\phi^{-1}$	SA	BWT	LF	$T_{\text{SA}[i]}$

0	C	6	14	18	C	4	$
1	T	11	8	7	C	5	ATATGTTGGTC$
2	A	7	9	2	T	11	ATGTCATATGTTGGTC$
3	T	13	10	9	T	12	ATGTTGGTC$
4	G	15	11	17	T	13	C$
5	T	16	13	6	T	14	CATATGTTGGTC$
6	C	17	0	0	S	0	CTATGTCATATGTTGGTC$
7	A	18	2	14	T	15	GGTC$
8	T	1	16	15	G	7	GTC$
9	A	2	17	4	T	16	GTCATATGTTGGTC$
10	T	3	12	11	T	17	GTTGGTC$
11	G	4	1	1	C	6	TATGTCATATGTTGGTC$
12	T	10	18	8	A	1	TATGTTGGTC$
13	T	5	3	16	G	8	TC$
14	G	0	15	5	G	9	TCATATGTTGGTC$
15	G	14	4	13	T	18	TGGTC$
16	T	8	5	3	A	2	TGTCATATGTTGGTC$
17	C	9	6	10	A	3	TGTTGGTC$
18	$	12	7	12	G	10	TTGGTC$

Search text *T* = “CTATGTCATATGTTGGTC$” with its ϕ mapping, $\phi^{-1}$ mapping, suffix array SA, Burrows-Wheeler transform BWT or L, mapping, and suffixes (where the first characters represent F). The ϕ, $\phi^{-1}$, and BWT runs are delineated by horizontal lines.

**Table 2:** Move table $M_{\text{LF}}$ corresponding to search text *T* = “CTATGTCATATGTTG-GTC$”.

j	c	p	π	ξ

0	C	0	4	1
1	T	2	11	6
2	$	6	0	0
3	T	7	15	9
4	G	8	7	3
5	T	9	16	10
6	C	11	6	2
7	A	12	1	0
8	G	13	8	4
9	T	15	18	11
10	A	16	2	1
11	G	18	10	5
12		19	19	12

**Table 3:** Move table $M_{\phi }$ for search text T = “CTATGTCATATGTTGGTC$”.Left: unbalanced, right: balanced.

j	p	π	ξ

0	0	6	4
1	l	11	5
2	2	7	4
3	3	13	7
4	4	15	9
5	8	1	1
6	12	10	5
7	13	5	4
8	l4	0	0
9	15	14	8
10	16	8	5
11	18	12	6
12	19	19	12

j	p	π	ξ

0	0	6	4
1	1	11	6
2	2	7	4
3	3	13	8
4	4	15	10
5	8	1	1
6	10	3	3
7	12	10	6
8	13	5	4
9	14	0	0
10	15	14	9
11	16	8	5
12	18	12	7
13	19	19	13

**Table 4:** Move table $M_{\text{LF}}^{\text{rev}}$ corresponding to search text $T^{\text{rev}}$ = “$CTGGTTGTATACTGTATC”.

j	c	p	π	ξ

0	C	0	4	1
1	T	1	11	6
2	$	5	0	0
3	A	6	1	1
4	T	7	15	9
5	G	10	7	4
6	A	11	2	1
7	G	12	8	4
8	A	14	3	1
9	C	15	5	2
10	T	16	18	12
11	C	17	6	3
12	G	18	10	5
13		19	19	13

**Table 5:** Move table $M_{\phi -1}$ for search text *T* = “CTATGTCATATGTTG-GTC$”. Left: unbalanced, right: balanced.

j	p	π	ξ

0	0	14	10
1	1	8	5
2	5	13	9
3	6	0	0
4	7	2	1
5	8	16	11
6	10	12	8
7	11	1	1
8	12	18	11
9	13	3	1
10	14	15	11
11	15	4	1
12	19	19	12

j	p	π	ξ

0	0	14	10
1	1	8	5
2	5	13	9
3	6	0	0
4	7	2	1
5	8	16	11
6	10	12	8
7	11	1	1
8	12	18	12
9	13	3	1
10	14	15	11
11	15	4	1
12	17	6	3
13	19	19	13

**Table 6:** Details of the pan-genomes that are used for benchmarking.

Dataset	#	$n/10^{6}$	$r/10^{6}$	$r^{\text{rev}}/10^{6}$	n/r
Human chrom. 19	2	111.61	31.16	31.16	3.58
	4	223.22	31.63	31.63	7.06
	8	446.44	32.00	32.00	13.95
	16	892.89	32.40	32.40	27.56
	32	1785.77	32.83	32.82	54.40
	64	3 571.53	33.34	33.34	107.12
	128	7143.04	34.05	34.05	209.76
	256	14286.12	35.62	35.62	401.07
	512	28 572.24	39.24	39.24	728.22

*E. coli*	2	10.08	5.06	5.06	1.99
	4	20.11	9.11	9.11	2.21
	8	40.16	11.43	11.43	3.51
	16	80.14	15.17	15.17	5.28
	32	158.30	19.42	19.42	8.15
	64	317.21	27.34	27.35	11.60
	128	630.15	34.68	34.68	18.17
	256	1262.28	52.06	52.06	24.24
	512	2 527.97	64.20	64.20	39.38
	1024	5 075.37	78.79	78.79	64.42
	2 048	10172.03	98.74	98.74	103.02
	3 264	16 207.62	118.15	118.15	137.18

For each pan-genome, we report the number of chromosomes or strains it contains, the length n of the corresponding concatenated string T, the number of runs r in the BWT of T, the number of runs $r^{\text{rev}}$ in the BWT of $T^{\text{rev}}$, and the ratio of n and r.

## Data Availability

The datasets supporting the conclusions of this article are publicly available, and the ‘Data and Hardware’ section lists all corresponding dataset identifiers and references. Details are available at https://github.com/biointec/b-move/tree/data/2024. The C++ source code of b-move is available at https://github.com/biointec/b-move under the GNU AGPL v3.0 license.

## Footnotes

1 Details available at https://github.com/biointec/b-move/tree/data/2024

2 Available at https://github.com/U-Ar/br-index

3 By Renders et al. [27, 28, 29, 26], available at https://github.com/biointec/columba

## List of abbreviations used

AGPL: Affero general public license
APM: Approximate pattern matching
BWA: Burrows-Wheeler aligner
BWT: Burrows-Wheeler transform
CIGAR: Compact Idiosyncratic Gapped Alignment Report
CPU: Central processing unit
FM: Ferragina and Manzini
GB: Gigabyte
GHz: Gigahertz
ISA: Inverse of the suffix array
LCP: Longest common prefix
LF: Last-to-First (property)
MEM: Maximal exact match
NCBI: National center for biotechnology information
PLCP: Permuted longest common prefix array
RAM: Random-access memory
RL: Run-length
SA: Suffix array
SAM: Sequence Alignment/Map format
WABI: Workshop on Algorithms in Bioinformatics
