# Supplementary material for: b-move: Faster Lossless Approximate Pattern Matching in a Run-Length Compressed Index
Source: Res Sq. 2024 Nov 18:rs.3.rs-5367343. Preprint. [Version 1] doi: 10.21203/rs.3.rs-5367343/v1 (PMC11601852; doi:10.21203/rs.3.rs-5367343/v1)
Supplement: Supplement 1 [file NIHPPRS5367343V1-supplement-1.pdf]

Supplementary Files

This is a list of supplementary files associated with this preprint. Click to download.

- AdditionalFile.pdf
